# Genomic analysis of prophages in 44 clinical strains of *Pseudomonas aeruginosa* isolated in Saudi Arabia

**DOI:** 10.3389/fcimb.2025.1563781

**Published:** 2025-04-28

**Authors:** Ahlam Alsaadi, Mohammed Imam, Abdulrahman A. Alghamdi, Safia S. Aljedani, Amal Alsari, Haya Aljami, Mohammad Bosaeed

**Affiliations:** ^1^ King Abdullah International Medical Research Center, King Saud Bin Abdulaziz University for Health Sciences, Riyadh, Saudi Arabia; ^2^ Department of Microbiology and Parasitology, Qunfudah College of Medicine, Umm Al-Qura University, Al-Qunfudah, Makkah, Saudi Arabia; ^3^ Department of Medicine, King Abdulaziz Medical City, Ministry of National Guard Health Affairs, Riyadh, Saudi Arabia

**Keywords:** *Pseudomonas aeruginosa*, prophages, multi-drug resistance, bacteriophages, Saudi Arabia

## Abstract

Prophages are bacteriophages that integrate their genomes into the bacterial chromosome. This research aimed to analyze and characterize prophages integrated into 44 *Pseudomonas aeruginosa* strains isolated from tertiary hospitals in Saudi Arabia. A total of 97 intact prophages were identified among clinical strains, with 16 prophages found present in more than one strain simultaneously. All prophages were found to have lengths ranging from 7.7 kb to 74.1 kb, and their GC content was found to be between 49.91% and 64.9%. Our findings show that prophages are present in the majority of the isolated *P. aeruginosa* strains (41 out of 44). Additionally, several proteins related to viral defense (toxin/antitoxin modules and proteins against restriction-modification enzymes) were identified, supporting the idea that prophages influence bacterial pathogenesis and anti-phage defenses.

## Backgrond

*Pseudomonas aeruginosa* is an opportunistic, Gram-negative pathogen and a member of the diverse and complex *Pseudomonas* genus (Pirnay et al., 2009). *P. aeruginosa* persists in both clinical and environmental settings and causes a wide spectrum of human infections, ranging from mild to life-threatening conditions (Diggle and Whiteley, 2020). The treatment of infections caused by *P. aeruginosa* presents one of the greatest therapeutic challenges (Pelegrin et al., 2021). Antibiotic resistance in *P. aeruginosa* can develop either through mutation that modify the expression or function of resistance-related genes or by acquiring resistance genes via mobile genetic elements (MGEs) (Silby et al., 2011). The accessory genome in *P. aeruginosa* consists mainly of mobile genetic elements such as plasmids, transposons, and temperate bacteriophages (prophages) (Silby et al., 2011). *P. aeruginosa* is listed in the ESKAPE group of pathogens, a designation for six bacteria recognized for critical clinical importance due to their increased antimicrobial-resistance rates (Rice, 2008).

Bacteriophages, or phages for short, are bacterial viruses considered obligate intracellular parasites. Phages are highly abundant on earth (Ackermann, 2007). During host infection, typically phages undergo one of two different life cycles: the lysogenic or the lytic cycle (Penadés et al., 2015; Williams et al., 2008). For phage therapy, strictly lytic phages, which directly undergo the lytic life cycle and lyse the target bacterial cells are preferable. Phages that follow the lysogenic life cycle integrate their genomes into the genome of their bacterial host, thereby contributing to bacterial fitness and bacterial genomic diversity, including the acquisition of antibiotic resistance genes and virulence factors (Gandon, 2016). Phages that integrate their genomes into the bacterial host are referred to as “prophages” or “temperate” phages (Gandon, 2016; Gordon et al., 2017). Recent studies have shown that prophages interact with their bacterial host’s regulatory cascade and interfere with the host’s immune system, in addition to encoding toxins, lytic proteins, and antimicrobial-resistance genes (Cieślik et al., 2021). For instance, in clinical *P. aeruginosa* prophage were found to code complete type II toxin-antitoxin module, exhibiting homology to the BrnT toxin and a CopG family antitoxin while other encode for complete type II TA system YoeB/YefM that is related to antibiotic resistance, biofilm formation, serum survival and host infection (Heaton et al., 2012; Ma et al., 2021). Moreover, the type II toxin YafO and a type IV antitoxin AbiEi were found in P. aeruginosa prophages all in which counteract bacterial defenses or to compete against external phages targeting the host (González de Aledo et al., 2023; Leroux and Laub, 2025). Therefore, the impact of prophages on human health and disease are largely unexplored and is still under investigation.

Early estimates found that prophages account for 10 to 20% of the host’s genome (Hatfull and Hendrix, 2011). The presence of prophages within the bacterial genome can provide the host with a selective advantage, for instance, the acquisition of prophages in clinical *P. aeruginosa* has been shown to reduce antibiotic susceptibility and enhance biofilm formation (Tariq et al., 2019). Among these, *P. aeruginosa* filamentous prophages Pf4 are highly upregulated and contributed to both biofilm formation and virulence (Hay and Lithgow, 2019; Platt et al., 2008; Rice et al., 2009). Additionally, prophages DMS3 and pp3 play distinct roles in host adaptation: DMS3 inhibits infection by phages that utilize the type IV pilus as a receptor, while pp3 promotes biofilm formation, facilitating bacterial persistence (Canchaya et al., 2004; Li et al., 2017; Little, 2005; Shah et al., 2021). Prophages can protect bacteria from environmental stressors and confer antibiotic resistance to bacterial cells by enhancing bacterial fitness through mechanisms such as, toxin-antitoxin systems, superinfection exclusion, biofilm formation and horizontal gene transfer (HGT) (Wang et al., 2010). These temperate phages significantly contribute to the extensive genomic diversity of bacteriophages through HGT and recombination (Dion et al., 2020).

Prophage-host interactions and dynamic adaptations over time have resulted in the acquisition of numerous defense mechanisms in response to selective pressures. These mechanisms including restriction-enzymes (RM) systems, clustered regularly interspaced short palindromic repeats (CRISPR) and CRISPR-associated (*cas*) genes, the abortive infection (Abi) systems, as well as the accumulation of a variety of mutations in surface receptor proteins (Ambroa et al., 2022; Labrie et al., 2010). As the evolutionary race between bacteria and their viral predators continue, new anti-viral mechanisms have been discovered and describes, such as the use of cyclic nucleotides as signaling molecules, such as the cyclic oligonucleotide-based antiphage signaling system, the pyrimidine cyclase system for antiphage resistance, and restriction by an adenosine deaminase acting on RNA (Cohen et al., 2019; Duncan-Lowey et al., 2023; Tal et al., 2021). Additionally, NAD+ depletion is a widespread bacterial defense response to viral infection (Garb et al., 2022; Tal and Sorek, 2022). As phages evolve, they have developed mechanisms such as anti-CRISPR (Acr) proteins and viral DNA methyltransferases. Acr proteins, first discovered in prophages infecting *P. aeruginosa* strains (Bondy-Denomy et al., 2013), are small peptides known to inhibit CRISPR-Cas activity. They function by binding to the different elements of the CRISPR machinery, thereby preventing DNA recognition, or inhibiting Cas protein activity once the protein complex has assembled around the target DNA (Pawluk et al., 2018).

Most *P. aeruginosa* strains have been identified to contain at least one prophage-like element; some are poly-lysogens, harboring several prophages in their genome (Knezevic et al., 2015; Silby et al., 2011). Lysogenic phages in *P. aeruginosa* have been shown to confer selective beneficial traits, such as O antigen conversion, biofilm development, and virulence (Hayashi et al., 1990; Kuzio and Kropinski, 1983; Rice et al., 2009). Additionally, prophages can serve as viable candidates for phage therapy, as they can be genetically engineered to be strictly lytic. For instance, in 2019, a patient with disseminated drug-resistant *Mycobacterium abscessus* was successfully treated with a cocktail of mutant phages engineered to eliminate lysogeny associated genes (Dedrick et al., 2019). Thus, identifying *P. aeruginosa* prophages provides valuable insight into the role of phages in *P. aeruginosa* fitness and pathogenicity.

Here, we investigate publicly available *P. aeruginosa* genomes from Saudi Arabia, which currently include 44 published clinical bacterial genomes, with a focus on temperate *P. aeruginosa* phages. This study aims to expand understanding of the nature, composition, and role of prophages found within a multicenter hospital *P. aeruginosa* strain collection from Saudi Arabia. Additionally, we analyze the genes these prophages harbor to overcome bacterial defenses and disseminate antibiotic resistance genes. Identifying prophages with no AMR or virulence factor genes in their genome is beneficial as they can hold potential as phage therapy candidates.

## Materials and methods

### Origin of *P. aeruginosa* isolated genomes

A total of 44 complete genomes of *P. aeruginosa* isolates from 44 patients were obtained from the National Center for Biotechnology Information (NCBI) database (https://www.ncbi.nlm.nih.gov/) (Last accessed on April 17, 2023). Demographic data of the patients were extracted from the original study’s **Supplementary Data** and presented in the study (see **Table 1**; **Supplementary Table 1**), bacterial genomes were sequenced using the MiSeq Illumina platform with a 2 x 300 bp paired-end reads protocol, as previously described (Doumith et al., 2022). The whole genome extraction and sequencing methods are described (Doumith et al., 2022), and genomes were deposited in Genebank under the accession numbers PRJNA751257.

**Table 1:** Characteristics of the 44 P*. aeruginosa* clinical strains.

Strain ID	MLST	β-lactamase	Origin	GenBank accession no.	Accession ID
RPA91	ST235	*bla*GES-15	Respiratory	DAHMLR000000000.1	SAMN20514488
RPA85	ST233	*bla*VIM-2, *bla*OXA-4	Respiratory	DAHMLQ000000000.1	SAMN20514487
RPA78	ST111	*bla*VIM-28	Urine	DAHMLP000000000.1	SAMN20514486
RPA66	ST235	*bla*VEB-16	Urine	DAHPTU000000000.1	SAMN20514485
RPA61	ST1076	*-*	Respiratory	DAHPTT000000000.1	SAMN20514484
RPA41	ST235	*bla*GES-5	Respiratory	DAHMLO000000000.1	SAMN20514483
RPA37	ST357	*-*	Blood	DAHMLN000000000.1	SAMN20514482
RPA32	ST235	*bla*GES-5	Urine	DAHMLM000000000.1	SAMN20514481
RPA23	ST235	*bla*GES-5	Blood	DAHMNH000000000.1	SAMN20514480
RPA226	ST357	*bla*OXA-10, *bla*VEB-9	Blood	DAHMMR000000000.1	SAMN20514479
RPA206	ST235	*bla*GES-5	Respiratory	DAHMMQ000000000.1	SAMN20514478
RPA185	ST235	*bla*GES-5	Respiratory	DAHMMP000000000.1	SAMN20514477
RPA135	ST357	*bla*VIM-2, *bla*OXA-10, *bla*VEB-9	Respiratory	DAHMLL000000000.1	SAMN20514476
RPA128	ST235	*bla*GES-5	Blood	DAHPTS000000000.1	SAMN20514475
RPA117	ST1659	*-*	Blood	DAHMMO000000000.1	SAMN20514474
RPA109	ST235	*bla*GES-5	Blood	DAHMNG000000000.1	SAMN20514473
RPA100	ST1076	*-*	Respiratory	DAHMNE000000000.1	SAMN20514472
RPA10	ST233	*bla*VIM-2, *bla*OXA-33	Respiratory	DAHPUN000000000.1	SAMN20514471
MPA91	ST773	*bla*NDM-1	Respiratory	DAHMMM000000000.1	SAMN20514470
MPA54	ST235	*bla*GES-5	Wound	DAHPTR000000000.1	SAMN20514469
MPA32	ST235	*bla*GES-5	Respiratory	DAHMNF000000000.1	SAMN20514468
MPA31	ST235	*bla*GES-5	Respiratory	DAHMLK000000000.1	SAMN20514467
MPA14	ST235	*bla*GES-5	Respiratory	DAHPTP000000000.1	SAMN20514466
MPA01	ST235	*bla*GES-5	Urine	DAHMMN000000000.1	SAMN20514465
JPAU94	ST235	*bla*GES-5	Urine	DAHPTO000000000.1	SAMN20514464
JPAU63	ST235	*bla*GES-5	Urine	DAHPTN000000000.1	SAMN20514463
JPAU54	ST308	*-*	Urine	DAHMLC000000000.1	SAMN20514462
JPAU51	ST1659	–	Urine	DAHMNB000000000.1	SAMN20514461
JPAU32	ST375	*bla*GES-5	Urine	DAHMMH000000000.1	SAMN20514460
JPAR79	ST235	*bla*GES-5	Respiratory	DAHMLJ000000000.1	SAMN20514459
JPAR65	ST235	*bla*GES-5	Respiratory	DAHPTM000000000.1	SAMN20514458
JPAR60	ST233	*bla*VIM-2, *bla*OXA-33, *bla*PER-1	Respiratory	DAHMNC000000000.1	SAMN20514457
JPAR31	ST235	*bla*GES-1	Respiratory	DAHPTL000000000.1	SAMN20514456
JPAR102	ST865	*-*	Respiratory	DAHPTK000000000.1	SAMN20514455
JPAO31	ST1020	*-*	Ear Swab	DAHMNA000000000.1	SAMN20514454
JPAB50	ST244	*bla*OXA-232	Blood	DAHPTY000000000.1	SAMN20514453
JPAB41	ST357	*bla*OXA-10, *bla*VEB-9	Blood	DAHPTX000000000.1	SAMN20514452
JPAB38	ST829	*-*	Blood	DAHPTV000000000.1	SAMN20514451
JPAB28	ST233	*bla*OXA-33, *bla*PER-1	Blood	DAHPTW000000000.1	SAMN20514450
JPAB24	ST2374	*-*	Blood	DAHMLB000000000.1	SAMN20514449
JPAB21	–	*-*	Blood	DAHMNI000000000.1	SAMN20514448
HPA69	ST500	*-*	Urine	DAHPTQ000000000.1	SAMN20514447
DPA57	ST357	*bla*VIM-2, *bla*OXA-10, *bla*VEB-9	Respiratory	DAHMND000000000.1	SAMN20514446
DPA39	ST641	*-*	Blood	DAHPTJ000000000.1	SAMN20514445

Carbapenem-resistant *P. aeruginosa* were isolated between March 2018 and April 2019 from five different hospitals located in the eastern, western and, central regions of Saudi Arabia and various sites of infection (Doumith et al., 2022).

### Identification of prophages in *P. aeruginosa* strains

Whole-genome sequences of *P. aeruginosa* clinical strains were used to identify and annotate prophages by submitting FASTA files via the web interface to PHASTER (PHAge Search Tool Enhanced Release) (https://phaster.ca/), a bioinformatics tool designed to rapidly identify and annotate putative prophage genomes sequences within the contigs of each bacterial genome. *P. aeruginosa* genomes were accessed on 23 04 2023. According to scoring criteria, PHASTER identifies prophages in three different categories: intact (score >90), questionable (score 70-90), and incomplete (score <70). Only intact prophages with scores of >90 were selected for this study. Additionally, PHASTER provides the location or insertion site of prophage within the bacterial genome, the number of coding DNA sequences (CDSs), and the GC% content of each prophage genome. Prophage genomes content were annotated using Prokka (https://github.com/tseemann/prokka) (last accessed on January 10, 2024) and the PHROGs database, which represents a library of families of different prokaryotic virus proteins generated using a new clustering approach based on remote homology detection, (https://phrogs.lmge.uca.fr/) (last accessed on January 10, 2024) (Terzian et al., 2021).

### *P. aeruginosa* prophage classification based on genomic similarity

With the increasing amount of phage genomic data, phages are now classified based on their genome similarity, as defined by the International Committee on the Taxonomy of Viruses (ICTV). Taxonomic classification of dsDNA phage genomes was conducted using a recently published automated high-throughput computational tool (taxmyPHAGE) (https://github.com/amillard/tax_myPHAGE/tree/main) designed for genus- and species-level identification of bacteriophages (Millard et al., 2024). In this study, we searched for closely related phages based on their genome similarity to classify prophage genomes using taxmyPHAGE. Input phage genomes were prophages whole genome sequences as FASTA format files, and the output data included information on the phage genus and species, along with similarity scores. Based on the tool guidelines, dsDNA phage genomes with an average nucleotide identity (ANI) ≥95% are considered the same species, and bacteriophages with an ANI ≥ 70% over 100% of the genome are considered to belong to the same genus (Millard et al., 2024). Also, Genome-based phylogeny and tree construction was performed using the VICTOR web platform to compare Psedumonas prophage genomes from this study with three members of the *Casadabanvirus* genus, as currently recognized by the ICTV (https://ictv.global) (Meier-Kolthoff and Göker, 2017). Whole-genome amino acid sequences comparisons were conducted using the Genome-BLAST Distance Phylogeny method (GBDP) approach, applying the d0 distance formula (Meier-Kolthoff et al., 2013). The resulting intergenomic distances were used to construct a balanced minimum evolution phylogenetic tree using FASTME 2.0, incorporating subtree pruning and regrafting (SPR) optimization and supported by 100 pseudo-bootstrap replicates (Lefort et al., 2015). The final tree was midpoint-rooted and visualized using the ggtree package (Yu et al., 2017). Species, genus, and family demarcation thresholds were determined using the OPTSIL clustering algorithm (Göker et al., 2009). Applying standard parameters and an F value of 0.5, protein homology was assessed using HHPRED (Zimmermann et al., 2018) and PHYRE2 (Kelley et al., 2015), while domain architecture predictions were carried out using the SMART tool (Letunic et al., 2021).

### Phylogenetic analysis of common *Pseudomonas* prophages

All identified complete prophage sequences were aligned using the MAFFT Version 7.0 program (https://maft.cbrc.jp/alignment/server/), using the strategy ‘auto’. A phylogenetic tree of the genomes was subsequently constructed using the phylogeny program (https://maft.cbrc.jp/alignment/server/phylogeny.html) using the neighbor-joining method using bootstrap values of 1000 replicates. The generated phylogenetic tree was visualized using the iTOL program (Baliga et al., 2021).

### Identification of virulence factors in prophage genomes

Prophage genomes were used to screen for virulence factor (VF) genes against the Virulence Factors of Pathogenic Bacteria Database (VFDB) (http://www.mgc.ac.cn/cgi-bin/VFs/v5/main.cgi, last accessed on November 20, 2023) (Liu et al., 2019). The VFDB was established in 2004 to provide up-to-date information about VFs from various bacterial pathogens and serves as a comprehensive repository system of bacterial virulence factors. Furthermore, VF genes were identified using VirulanceFinder 2.0 (https://cge.cbs.dtu.dk/services/VirulenceFinder/, last accessed on November 20, 2023).

### Identification of antibiotic resistance genes in prophage genomes

All prophage genomes were screened for the presence of antibiotic resistance genes in the Comprehensive Antibiotic Resistance Database (CARD) (https://card.mcmaster.ca/, last accessed on November 20, 2023) (Alcock et al., 2020). CARD is a biological database integrating molecular and sequence data while collecting resistance determinants and associated antibiotics. Resistance genes were also assessed by screening for AMR genes using the ResFinder 4.1 database (https://cge.cbs.dtu.dk/services/ResFinder-4.1/, last accessed on November 20, 2023).

### Statistical analysis

The Pearson’s correlation coefficient (r) analysis was conducted using DATAtab: Online Statistics Calculator (DATAtab: DATAtabTeam, 2025) https://datatab.net. Pearson’s correlation was computed with a two-tailed test, and a threshold of p < 0.05 was considered statistically significant. Additionally, scatter plots were generated using DATAtab’s online tool, which provided visual representation of the relationships between variables. All statistical assumptions, including normality, were verified before analysis.

## Results

### Abundance of prophages in *P. aeruginosa* genomes

**Figure 1:**
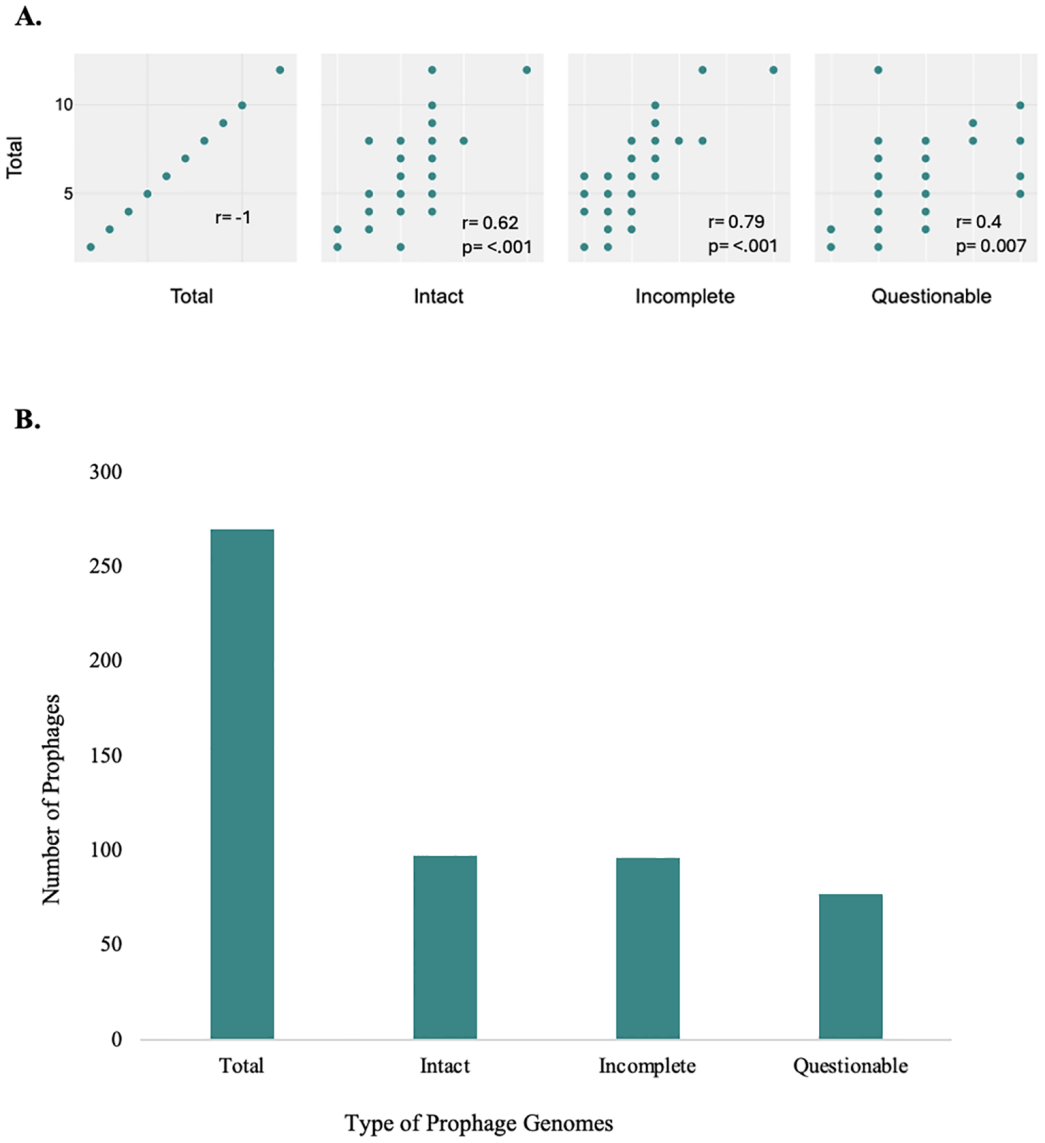
Prophage distribution in *P. aeruginosa* genomes. Illustration of the correlation coefficients between the total number of intact, incomplete, and questionable prophages **(A)**, and total number of prophages in *P. aeruginosa* genomes, categorized as intact, incomplete, and questionable **(B)**. Prophages were detected and classified using the PHASTER with default arguments and its scoring system. **(A)** Correlation and scatter plot of prophage genome types and their distribution. The analysis reveals a significant correlation between the total category and all three subcategories, as the strongest relationship is with Incomplete, followed by Intact, while the weakest is with Questionable, all with p-values <0.05; these correlations are statistically significant. **(B)** There were differences in the distribution of prophages of varying completeness on the chromosomes of *P. aeruginosa*. The integration of intact prophages in the bacterial genome indicates a recent infection with a temperate phage (Costa et al., 2018). There is a significantly high prevalence of defective phages (**Figure 1**, **Supplementary Table 2**).

The genome sequences of 44 P*. aeruginosa* clinical isolates from 44 patients were screened for prophages. A total of 270 prophage-like elements were detected (**Supplementary Table 2, 9**), of which 97 were classified as intact, 96 as incomplete, and 77 as questionable (**Figure 1**). The number of intact bacteriophages found in each genome ranged from one to six, with a median of two, adding up to a final value of 97 prophages (**Supplementary Table 2**). The correlation between the prophage genome type and its distribution was investigated. The Pearson’s correlation coefficients (R-values) were Total & Incomplete (r = 0.79, p < 0.001), Total & Intact (r = 0.62, p < 0.001) and Total & Questionable (r = 0.4, p = 0.007). This correlation reveals a significant correlation between the total category and all three subcategories, as the strongest relationship is with Incomplete, followed by Intact, while the weakest is with Questionable. The R-values for all categories were greater than 0.5, indicating a positive correlation between increased the total prophages count with intact and incomplete prophage count increase. In total, 41 P*. aeruginosa* clinical (93.1%) were found to harbor at least one to six prophages. However, in strains JPAB41, JPAU51, and RPA226, no intact prophages were found. This indicates that prophages are abundant in the genomes of *P. aeruginosa*.

### AMR and virulence factors found in prophages

Prophages provide genomic plasticity and host adaptation for their bacteria (Shen et al., 2020), and act as important vehicles carrying virulence factor (VF) and antimicrobial resistance (AMR) genes (Colomer-Lluch et al., 2011). Among all prophages, only the prophage genome AA67 was found to carry a chloramphenicol resistance gene catB7_1 detected by ResFinder (**Supplementary Table 10**). There were no VF or AMR genes identified in other intact prophages in this study, this absence may be attributed to a complex decay process involving the accumulation of mutations, deletions or genetic rearrangements, leading to the loss of non-essential genes such as AMR genes. Additionally, carrying AMR genes can impose a metabolic burden on bacteria, especially in antibiotics-free environments, potentially reducing bacterial fitness. Thus, this selective pressure may favor retention of prophages lacking these genes (Kondo et al., 2021). However, VF genes were detected in the incomplete genomes of JPAO31R1in, JPAO31R7in, JPAR31R6in, JPAU63R6in, RPA10R10in, RPA41R7in, RPA109R8in, RPA128R6in, RPA135R4in, and MPA31R8in (**Supplementary Table 3**). In the clinical *P. aeruginosa* isolates, the presence of VF and/or AMR genes in incomplete prophages rather than intact prophages could be often associated with that intact prophages often retain genes essential for phage-related functions, whereas incomplete prophages may accumulate genes advantageous to bacterial survival. In the study by Kondo et al. (2021), they observed that AMR genes were frequently located near recombination-related genes, such as integrases and transposases, within prophage regions. In contrast, VF genes were less commonly associated with these recombination-related genes. The distinct distribution patterns imply that AMR and VF genes may be acquired and propagated through different evolutionary pathways within bacterial genomes (Kondo et al., 2021). In the genomes of nine different incomplete prophages, an annotated *Escherichia coli iss* gene was detected with 97.41% coverage (GenBank accession no. AF042279). The *iss2* gene contributes to bacterial survival and is not usually found in *Pseudomonas* spp., suggesting acquisition through horizontal gene transfer. The incomplete prophage genome JPAO31R1in was found to carry eight different VF genes, including *algL*, *algX*, *algG*, *algE*, *algK*, *alg44*, *alg8*, and *algD*). Some of these alginates represent key exopolysaccharides involved in biofilm formation and alginate production by *P. aeruginosa* (Monday and Schiller, 1996).

### Genetic diversity of *P. aeruginosa* prophages

**Figure 2:**
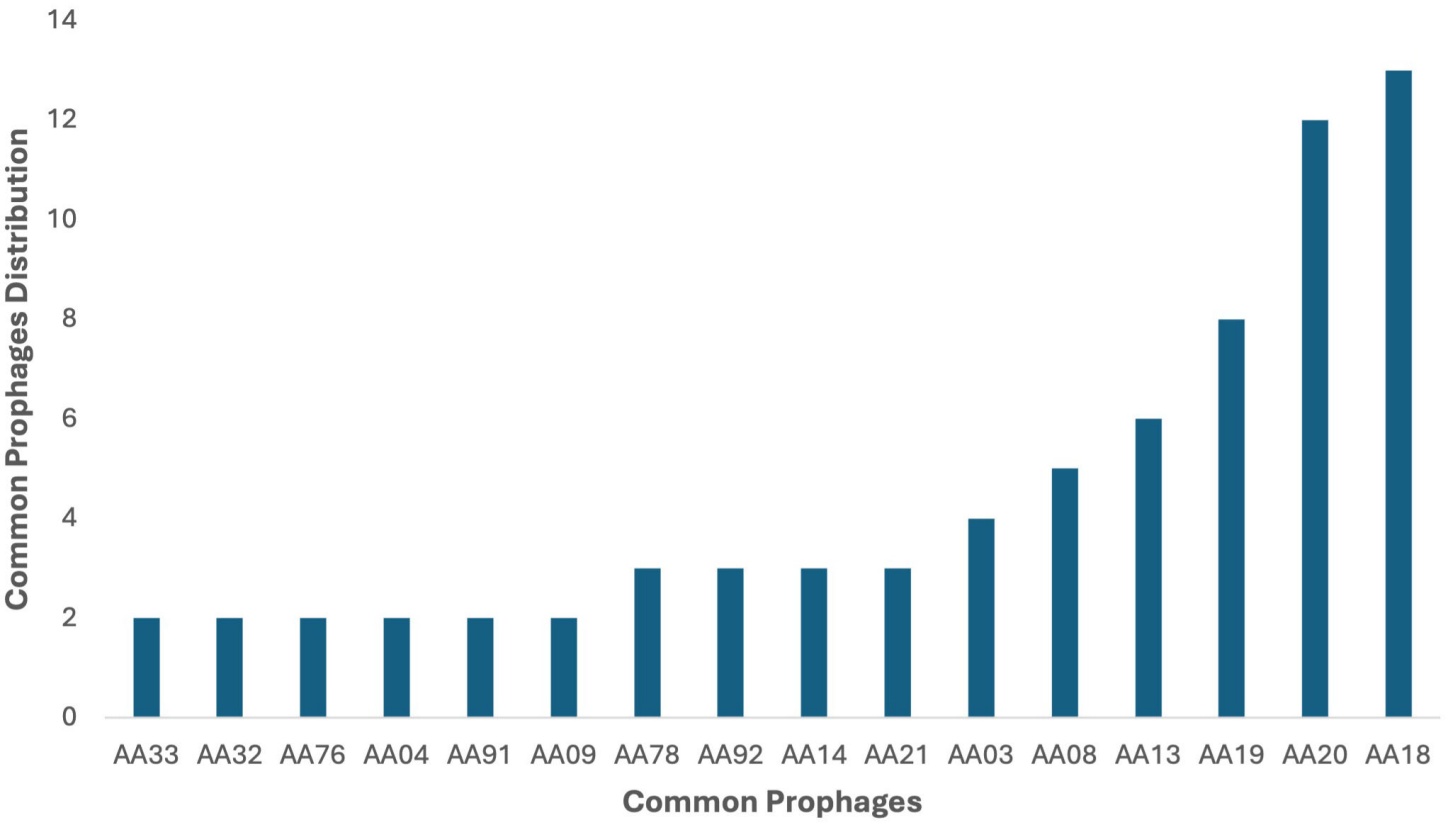
Distribution of intact prophages in the 44 P*. aeruginosa* strains. Among the identified prophages, AA18 was the dominant intact prophage in 13 P*. aeruginosa* genomes in the study, followed by AA20 in 12 genomes and AA19 in eight genomes. Prophages AA13, AA08, and AA03 were present in six, five, and four genomes, respectively. Four prophages were identified in three genomes each, while six other prophages were found in two genomes each.

Among the identified prophages, some prophages were found to be present in more than one clinical strain, with 13 P*. aeruginosa* genomes carrying the prophage AA18, 12 genomes harboring AA20 as the second most abundant prophage, and eight *P. aeruginosa* genomes carrying prophage AA19. The genomes of prophages AA13, AA08, and AA03 were found in six, five and four *P. aeruginosa* genomes, respectively (**Figure 2**). Four prophage genomes were present in three different genomes. In comparison, the other six common prophage genomes were only found twice in each bacterial genome (**Supplementary Table 4**; **Figure 2**). These common prophage genomes were distributed across a variety of strains with different sequence types and also had high percentages of integration on the chromosome. All prophage genome sizes ranged from 7.7 kb to 74.1 kb, and their GC% content was found to be between 49.91% and 64.9%, which is slightly lower than the host’s GC content of 65-67% for *P. aeruginosa* (Klockgether et al., 2011) (**Supplementary Table 5**). The differences in GC content among prophages are considered an evolutionary trait, indicative of the recent acquisition of prophage regions with exogenous origins (Fortier and Sekulovic, 2013). The most common prophage among *P. aeruginosa* strains, AA18, with a GC content of 62.67% indicates its adaptation to its host; the more similar the GC content of a prophage is to its host, the better adapted the prophage is. Additionally, prophages with shorter genome lengths present higher GC content, such as prophages AA29, AA47, AA67, and AA5 (**Supplementary Table 5**). It is important to note that the genomes were divided into contigs, which implies that PHASTER may have underestimated the correct number of intact prophages. Some prophages may have split into different contigs and thus identified as incomplete or questionable prophages.

### Analysis of prophage distribution according to taxonomic classification and genome size

**Figure 3:**
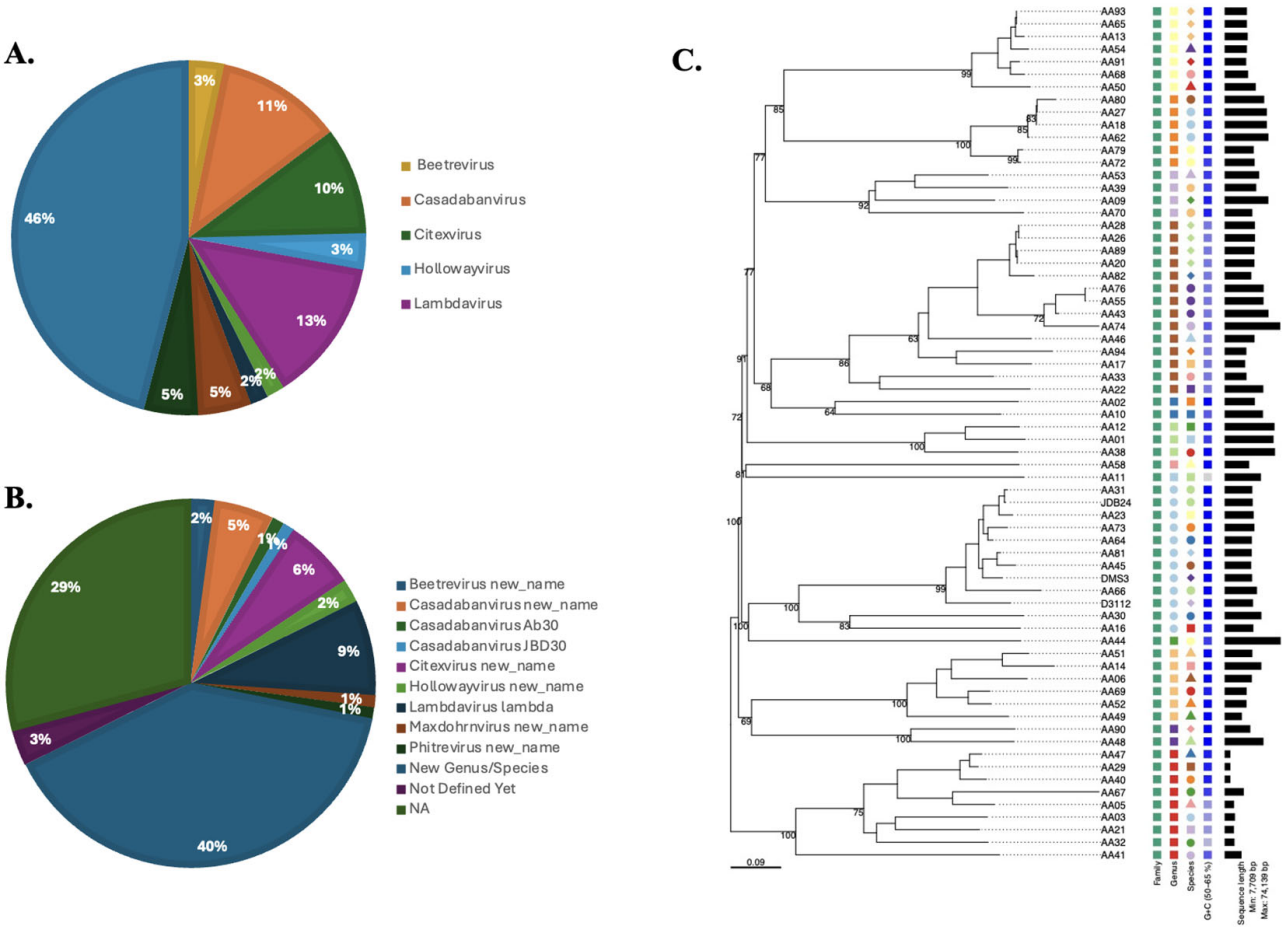
Taxonomic classification of prophage genomes. TaxmyPHAGE classified 97 prophage genomes, identifying 28.1% as belonging to known genera, and 71.8% as novel and unknown genera. Prominent taxa included *Casadabanvirus* (7.2%) and *Lambdavirus* (8.3%), while less frequent taxa, such as *Beetrevirus* (2.08%), were observed at both the genus **(A)** and species **(B)** levels. **(C)** Phylogeny of *Pseudomonas* prophages in current study and three different prophages (JDB24 (NC 020203), DMS3 (NC 008717), and D3112 (NC 005178) within the *Casadabanvirus* genus as listed by ICTV. The tree was inferred from the amino acid sequences using the VICTOR pipeline with the GBDP d0 formula.

Using TaxmyPHAGE, 97 prophage genomes were classified into distinct taxonomic groups (**Supplementary Table 6**). TaxmyPHAGE identified the genera and species of the input prophage genome sequences; genomes were found to differ based on their genomic analysis (**Figure 3**). Genomic comparisons revealed that 28.1% of the genomes were from known genera, while 71.8% were classified to be from novel and unknown genera, 39.58% and 32.29% respectively. The majority of phages belong to novel or uncharacterized taxa at both the genus and species levels, suggesting the discovery of new phage groups. From the genera and species contributed significantly, like *Casadabanvirus* (7.2%), *Lambdavirus* (8.3%), and *Citexvirus* (6.25%). While genera and species such as *Beetrevirus* (2.08%) and *Hollowayvirus* (2.08%) were less common, suggesting they may be less widespread or specialized. Unexpectedly, the presence of *Lambdavirus* lambda was reported among *Pseudomonas* phages, in prophages genomes AA29, AA52, and AA62. These genome sequence annotations were verified using BLAST against the NCBI database to ensure accuracy, that the prophage genome annotations indicated *Lambdavirus* lambda. The results show a high degree of prophage diversity among the *P. aeruginosa* clinical strains. The average genome size of the prophages was 41.3kb, which is consistent with values reported from other studies working on different bacteria (Bobay et al., 2014; Canchaya et al., 2003).

### Phylogenetic relationship between prophages

**Figure 4:**
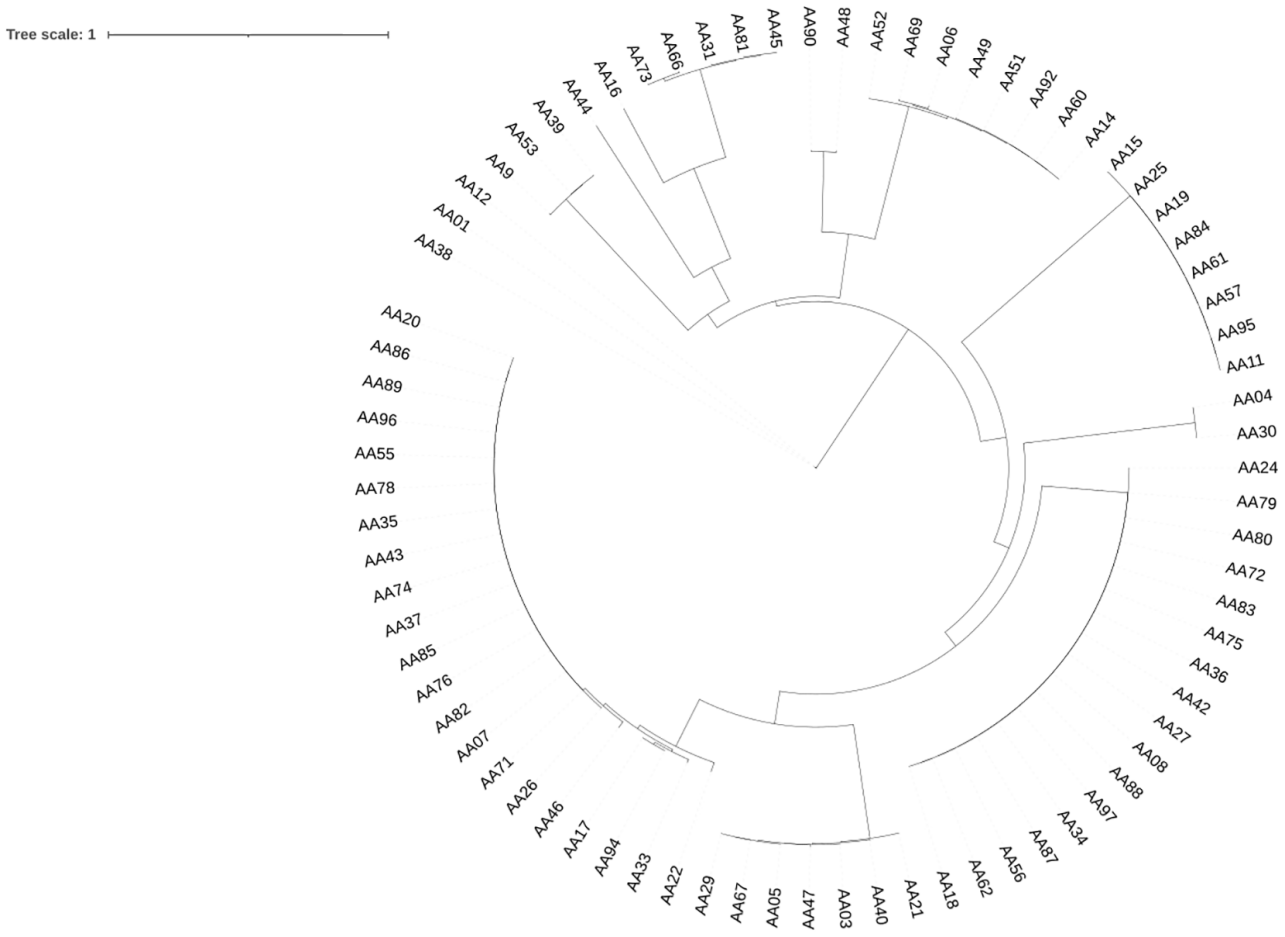
Phylogenetic Tree of *P. aeruginosa*-associated prophages. The phylogeny tree was constructed using the neighbor-joining method with the MAFFT program based on major capsid protein (MCP) sequences. Only prophage genomes containing MCP sequences were included in the analysis, while prophages without MCPs were excluded. The schematic representation of the tree was visualized using the iTOL v6 software (Letunic and Bork, 2021).

A phylogenetic tree was generated based on complete prophage sequences to evaluate diversity. The tree was constructed using the major capsid protein (MCP) as a reference (**Figure 4**). The phylogenetic analysis revealed that the prophages clustered according to their morphological family types, indicating that the predicted virion morphotypes correlate with the genomic phylogeny. This finding aligns with the new classification system of the International Committee on the Taxonomy of Viruses, which is based on genome and proteome data (Adriaenssens and Rodney Brister, 2017). Furthermore, phylogenetic analysis of the *P. aeruginosa* prophages investigated demonstrated a high diversity of evolutionary groupings and provided potential functional insights derived from the MCP sequences. Prophage genomes such as AA01, AA12, and AA09 are closely clustered, indicating lower genetic divergence and suggesting that their capsid structures may be highly conserved, which could imply a specific phage-host interaction. In contrast, prophages AA44, AA16, and AA73 share an evolutionary history, while the longer branches of AA45, AA09 with AA49, AA06, and AA92 suggest more distant relationships. These insights provide a framework for future studies on the diverse structural and functional roles of MCP in prophage adaptation. The tree was visualized using Interactive Tree of Life (iTOL) v6.

### Sequence type distribution

**Figure 5:**
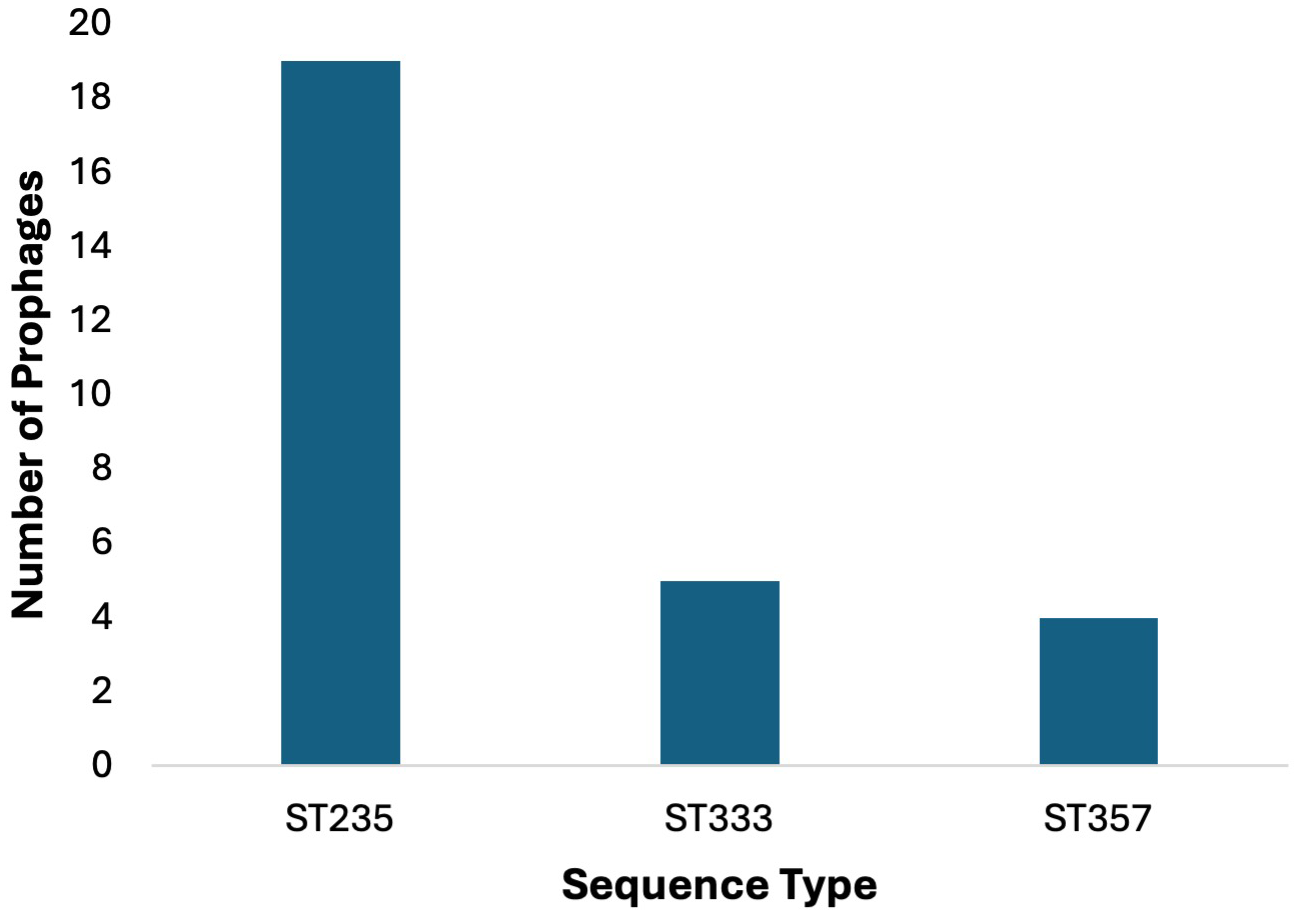
Prophage counts in most common sequence types (STs). Prophages were predominantly identified in the 44 P*. aeruginosa* strains belonging to ST235 (n=19), followed by ST233 (n = 5) and ST357 (n = 4).

In the sequence set consisting of 44 strains of *P. aeruginosa*, all strains had known sequence types (ST), except for one strain JPAB21 (**Table 1**), which were divided into 16 distinct types. The correlation between the *P. aeruginosa* ST and prophage harboring was studied (**Supplementary Table 7**). In the collection of 44 P*. aeruginosa* strains, ST235 was found to be the most prevalent type (n = 19), followed by ST233 (n = 5) and ST357 (n = 4), (**Figure 5**).

In ST235, prophage AA18 was found in 13 out of 19 strains, prophage AA20 in 12 out of 19, and prophage AA19 in 5 out of 19 strains; in ST233 2 out of 4, and in ST111, 1 out of 1 strain. *P. aeruginosa* strains with ST235 harbor the three most common prophages, which carry the GES-5 and GES-15 β-lactamases. The other three *P. aeruginosa* strains JPAB41, JPAU51, and RPA226, which did not harbor any of the three most common prophages, carried the GES-1 and GES-5 β-lactamases. In other ST, the distribution of these prophages was rare or even absent. This suggests that ST may relate to the types of prophages integrated into the chromosome.

The strains belonging to the different STs were more diverse in their prophage arrangement. Although the remaining strains harbored at least one intact prophage, the distribution of these prophages was irregular and lacked a clear association.

### Most common anti-phage defense systems identified in analyzed genomes

#### a. Defense against restriction-modification systems

In this study, 28 out of 97 prophages were found to encode DNA methyltransferases. Prophages use DNA methyltransferases to methylate their DNA to evade the host cell’s restriction-modification system, regulate viral gene expression, and facilitate DNA packaging into the preformed capsids (Burke et al., 2021; Loenen and Raleigh, 2014). Additionally, restriction alleviation proteins were found in 20 prophage genomes; these proteins are known to protect against the host cell’s restriction-modification systems (**Supplementary Table 8**) (Loenent and Murray1, 1986; Toothmant, 1981).

#### b. Toxin/antitoxin systems

Toxin-antitoxin (TA) modules play important roles in plasmid and prophage stability and are also key factors in bacterial physiology (Harms et al., 2018). A proposed system protects bacteria from phages, alongside CRISPR and restriction-modification systems. In this context, it is not surprising that prophages carry antitoxins to counteract bacterial defense mechanisms, or even toxins alone, to compete against external phages preying on their host (Leroux and Laub, 2022). In this study where the proposed system was detailed, prophages were found to encode TA modules belonging to the type II systems. The type II toxin YafO was found in 16 prophage genomes (Zhang et al., 2009). Additionally, the type II TA system putative toxin HigB2 was found in ten prophages. In bacteria, toxin HigB2 has been shown to reduce the expression of virulence-associated traits such as the production of pyochelin and pyocyanin, biofilm formation, and swarming motility. Thus, this system affects the pathogenicity of the strain in a manner not previously demonstrated for TA systems (**Table 2**; **Supplementary Table 8**) (Wood and Wood, 2016).

**Table 2:** Viral defense and regulatory proteins identified in 13 prophages common across all 44 P. *aeruginosa* genomes analyzed in this study.

Prophage	Viral Defense proteins				Regulatory Proteins	
	Glycosyltransferases and Acetylases	Defense Against Restriction- Modification Systems	TA systems	DNA Scission Proteins	Latency- Promoting Repressors	Other Proteins
AA18	ND	ND	Putative toxin HigB2	ND	ND	HTH-type transcriptional regulator PrtR
AA23	ND	ND	ND	ND	ND	ND
AA27	ND	ND	Putative toxin HigB2	ND	ND	HTH-type transcriptional regulator PrtR
AA34	ND	ND	Putative toxin HigB2	ND	ND	HTH-type transcriptional regulator PrtR
AA36	ND	ND	Putative toxin HigB2	ND	ND	HTH-type transcriptional regulator PrtR
AA42	ND	ND	Putative toxin HigB2	ND	ND	HTH-type transcriptional regulator PrtR
AA56	ND	ND	ND	ND	ND	ND
AA62	ND	ND	Putative toxin HigB2	ND	ND	HTH-type transcriptional regulator PrtR
AA75	ND	ND	Putative toxin HigB2	ND	ND	HTH-type transcriptional regulator PrtR
AA77	ND	ND	ND	ND	ND	ND
AA80	ND	ND	ND	ND	ND	HTH-type transcriptional regulator PrtR
AA83	ND	ND	Putative toxin HigB2	ND	ND	HTH-type transcriptional regulator PrtR
AA88	ND	ND	Putative toxin HigB2	ND	ND	HTH-type transcriptional regulator PrtR

*ND refers to No Data; no available information for the given parameter.

### DNA scission proteins

Prophages encode junction-resolving enzymes, such as Holliday junction resolvases. Specifically, prophages encode RusA-like Holliday junction resolvase, Lar-like restriction alleviation protein, and restriction alleviation Ral (**Supplementary Table 8**). These enzymes have been described as being involved in the degradation of the host’s DNA, self-DNA maturation, and cleavage prior to packaging (Wyatt and West, 2014).

## Discussion

This study encompassed the search and analysis of prophages within a set of 44 multidrug-resistant (MDR) *P. aeruginosa* clinical strains isolated from different hospitals across different regions of Saudi Arabia (Doumith et al., 2022). Our findings demonstrate that these prophages are present in the majority of strains (**Supplementary Tables 1** and **9**). Many of the prophages were found in more than one strain, following a similar ST pattern. In only 6.8% of the strains (n = 3), no intact prophages as predicted by the PHASTER tool were identified, showing that prophage harboring is a very frequent trait among circulating *P. aeruginosa* strains in Saudi Arabia.

Prophages have been linked to bacterial diversification and evolution, exerting a strong selection pressure on bacterial fitness and virulence (Brüssow et al., 2004; Canchaya et al., 2004; Casjens, 2003; Desiere et al., 2001). A limited number of studies have characterized the prevalence of prophages in bacterial species and evaluated their role in virulence in the Arab region, specifically in clinical strains from Saudi Arabia. In this study, we report the analysis of prophage prevalence in clinical MDR *P. aeruginosa* and discuss their possible contribution to the evolution of the pathogenicity of *P. aeruginosa*.

We found that the 44 P. *aeruginosa* strains harbored a total of 270 prophages following genomic analysis. A significant number of these prophages (n = 97) were classified as intact prophages, while detective prophages (96 incomplete and 77 questionable) were also detected in large numbers. The subset of 97 intact prophages were classified into distinct taxonomic groups (**Figure 3**). The defective prophages were attributed to strong stress selection, which resulted in mutations or gene loss that inactivated the prophages (Bobay et al., 2014). Genomic diversification was observed among the sequences, revealing novel genera and species among the prophages. Using the TaxMYPHAGE tool, genomic comparisons indicated that 33.33% of the prophages within *P. aeruginosa* genomes were assigned to known genera (**Figure 3**), while 55% were classified as novel genera. This highlights the discovery of new phage groups and emphasizes the need for additional research to better understand phage diversity within clinically isolated *P. aeruginosa* strains. Interestingly, prophage genomes AA29, AA52, and AA62 were classified under the *Lambdavirus* genus, however, when compared against the NCBI database, they unexpectedly showed similarities to *Pseudomonas* associated phages. This could be attributed due to two key reasons; (i) complex evolutionary and taxonomic relationships, such as horizontal gene transfer (Hulin et al., 2023); (ii) mis-annotation due to sequence similarities, which may also explain the inclusion of Lambdaviruses among Pseudomonas phages as classification tools rely heavily on conserved genomic regions. Moreover, as genomic technologies advance even more rapidly, that there will be more discoveries in the near future. These findings suggest exercising caution when classifying phages, as reliance on specific conserved regions may lead to inaccuracies in taxonomic assignments. Overall, our findings reveal a significant degree of genomic diversity within the prophages within the clinically isolated *P. aeruginosa* strains.

The correlation between bacterial genome sizes and their GC content was studied by Chen et al. (2016). Applying their findings to prophages, shorter prophage genomes might have higher GC content due to selective pressures favoring energy-efficient nucleotide usage and the retention of stable, essential genes. However, it is important to note that this is a hypothesis derived from related studies and this relationship is influenced by various factors, including the specific bacterial host, environmental conditions, and evolutionary pressures.

Some genes expressed from prophage regions can alter the properties of the host, ranging from increased protection against further phage infection to increased virulence (Casjens, 2003). High-risk STs, such as ST235, were identified as carrying various prophages. ST235 is known for its ability to acquire mobile genetic elements, its elevated antimicrobial resistance rates, and its global distribution (Kabic et al., 2023; Treepong et al., 2018). In particular, ST235, the most prevalent ST among MDR *P. aeruginosa* clinical isolates (**Table 1**). has been shown to lack a functional CRISPR-Cas system, thus explaining its ability to acquire exogenous genetic elements, such as genomic matter from bacteriophage.

The prevalence of the putative toxin HigB2 was highlighted, as a part of a TA system in prophage genomes, which is known to stabilize the prophage within the bacterial cell. Under stressful conditions, the activation of HigB2 might initiate entering a state of dormancy, allowing bacterial cells to withstand adverse environments and delay prophages from entering the lytic cycle. The dormant state may contribute to the persistence of bacteria even in the presence of antibiotics (Leroux and Laub, 2022; Yang and Walsh, 2017). Collectively, these findings highlight the functional versatility of prophage-encoded toxins like HigB2, underscoring their roles in bacterial survival and adaptation in challenging environments.

The prophage-encoded HTH-type transcriptional regulator PrtR acts as a key modulator in controlling phage life cycle transitions, responding to host stress, and potentially influencing host virulence. Its regulatory function ensures that the prophage remains latent under stable conditions and activates lytic genes when the host environment becomes unfavorable, enhancing both phage survival and bacterial adaptability (Dodd et al., 2005; Oppenheim et al., 2005). In the presence of DNA damage, such as treatment with mitomycin C, the activation of RecA causes the PrtR repressor to self-cleave, which together with PrtN, regulates the production of pyocins (Matsui et al., 1993; Penterman et al., 2014; Walker, 1984; Wu and Jin, 2005). This highlights the importance of prophage-borne counter-defense mechanisms, which not only protect the prophage against the bacterial host’s immune system, but also protect the host from infection by other phages, enabling survival and transmission to bacterial progeny.

Prophages can be induced from their host and can contribute to phage therapy. Although prophages incorporate their genomes into the host and integrate within the host genome, they can also serve as effective candidates for phage therapy. Genetic engineering of prophages, such as the removal of the integrase protein, and genomes are cleared of virulence genes, resistance related genes, and generally undesirable genes can make engineered prophages safe for use it patients. In 2019, a patient with a disseminated multi-drug-resistant *Mycobacterium abscessus* was treated with a cocktail of mutant phages engineered to remove lysogeny associated genes (Dedrick et al., 2019). This case highlighted the feasibility of phage engineering for complex bacterial infections. Another form of phage engineering involves extracting specific lytic proteins, such as lysins and holin, and using them for therapeutic purposes instead of whole viral particles. Their effect of these phage derived enzymes on MDR *P. aeruginosa* has been investigated *in vitro* and reported in the literature (Cui et al., 2023). Building on the prophages identified in this study, these approaches could be adapted in the future to design tailored therapies for *Pseudomonas*-related infections.

On the other hand, the presence of prophages can have an impact on antibiotic and phage therapies. Certain antibiotics can induce prophages within bacterial genomes, leading to the release of phage particles and potential horizontal gene transfer. A study demonstrated that common oral medications, including antibiotics, can lead to prophage induction in gut bacterial isolates, suggesting that antibiotic therapy might inadvertently activate prophages, influencing bacterial behavior and resistance patterns (Sutcliffe et al., 2021). Additionally, Prophages can confer resistance to superinfection by other phages through mechanisms such as superinfection exclusion and the CRISPR-Cas system. This resistance poses challenges to phage therapy, as therapeutic phages may be rendered ineffective against lysogenic bacteria (Bondy-Denomy et al., 2016).

One limitation of our study is that some of the *P. aeruginosa* isolates were sequenced using short-read bridge amplification technology (Illumina, Oxford Genomics Centre, Oxford, UK), generating 150 bp fragments. After assembly, these sequences resulted in 206 to 3,252 contigs per genome (an average of 1,602 contigs per genome). The more fragmented the genomes are, the more difficult it is to identify intact prophages, meaning that some prophages would have not been identified based on the tools used as they would have been split across several contigs. To circumvent this issue, a combination of both short- and long-read sequencing could be performed to obtain high-quality, complete bacterial genomes. This study focused its analysis on intact prophages only. Overall, our results suggest a significant contribution of prophages to the evolution and adaptation of *P. aeruginosa* clinical strains.

In conclusion, this study demonstrates the pivotal role of prophages in shaping the genomic landscape and adaptive capabilities of MDR *P. aeruginosa* clinical strains. These findings not only advance our understanding of phage-bacteria interactions but also open new avenues for therapeutic applications and treatment of MDR infections. Future research should focus on integrating long-read sequencing technologies with functional studies to enhance the therapeutic potential of prophages. This will help refine prophage classification, uncover novel targets, and develop customized interventions against *P. aeruginosa* and other multidrug-resistant pathogens.

## Data Availability

The original contributions presented in the study are included in the article/**Supplementary Material**. Further inquiries can be directed to the corresponding author.
